# “If you want to go fast, go alone. If you want to go far, go together”: A call for a Nordic engagement in the development of family medicine in Africa

**DOI:** 10.3109/02813432.2013.838340

**Published:** 2013-12

**Authors:** Per Kallestrup, Michael Schriver, Morten Lindbaek, Per Hjortdahl

**Affiliations:** Center for Global Health, Department of Public Health, Aarhus University, Aarhus, Denmark, Skødstrup Lægepraksis, Skødstrup, Denmark. E-mail: kallestrup@dadlnet.dk; Center for Global Health, Department of Public Health, Aarhus University, Aarhus, Denmark; Antibiotic Centre for Primary Care, Department of Family Medicine, Institute of Health and Society, Medical Faculty, University of Oslo, Oslo, Norway; Department of Family Medicine, Institute of Health and Society, Medical Faculty, University of Oslo, Oslo, Norway

## Introduction

In Scandinavia, general practice as a specialty in its own right developed over centuries. Milestones in the past 50 years are the foundations of professional associations and university-based research departments, requirements for postgraduate education, and full recognition as a specialty [1].

In many countries in Africa, the continent facing the world's greatest burden of disease with the fewest resources to tackle it, the specialty is in its childhood, infancy, or even barely conceived. In addition to resource constraints and structural obstacles, many countries face opposition from within the medical profession itself, where emphasis is put on vertical specialties.

Africa has an immense shortage of health professionals and with few exceptions physician clinicians are based in hospitals [2]. Family medicine networks in Africa advocate for engaging family physicians as leaders among equals in primary care teams in order to build an appropriate skill mix that can respond to the health care needs of the communities. Continuous training and team work are fundamentals for this development, as well as scale-up of the family medicine specialty [3].

## Examples of Nordic family medicine activities in Africa

In Botswana in the 1980s Norwegian family physicians played a major role in building the primary care system. Around 50 Norwegian GPs worked for 2–6 years as District Medical Officers with responsibility for large districts with 30–60 000 people. The system was built on specially educated nurses as primary care “doctors”.

In Rwanda, the first generation of six family physicians graduated in 2012. Their role in the public health system, as well as recognition, is yet to be determined. Having learned core competences in community orientation, comprehensive care, and clinical leadership, these specialists have important prerequisites to strengthen a patient-centred approach to health care in their districts. The Danish College of General Practitioners is supporting the further education of family physicians in Rwanda through clinical mentoring under the family medicine curriculum at the National University of Rwanda. The programme is initiated under “Partners in Practice” – an International Development Programme of the Danish College of General Practitioners [4], which recruits Danish GPs to partake in projects to support and capacity-build emerging family medicine institutions in partner countries.

In Malawi, three Norwegian Universities (Oslo, Bergen, Tromsø) are aspiring to become involved as key facilitators of the development of family medicine. This project is designed to support the development of the academic discipline of family medicine, in order to improve the quality of and access to primary care for the people of Malawi, particularly by establishing appropriate, district-based specialty training in family medicine for doctors, as well as facilitating the development of an academic Department of Family Medicine [5].

During the last few years the Ministry of Health in Norway, the University of Gezira, Sudan, and the Department of Global Public Health and Primary Care, University of Bergen, have collaborated on a project aimed at educating specialists in family medicine. So far about 200 local doctors are enrolled in this novel two-year master's programme, which relies heavily on web-based teaching and communication [3,6].

## New possibilities for mutual involvement and development

Primary health care has long been a solid backbone of health systems in Scandinavia, and we can be proud of this. Universal health coverage is an essential global health objective of our century [7], and on a global scale Scandinavia is a robust example of this. Scandinavia has experience worth sharing, and a lot to learn. Facing shortages of health professionals ourselves we must prepare coming generations to engage in effective health care teams and respond critically, constructively, and creatively to the existing system [8]. Also, serious economic cutbacks may make it relevant to learn from less privileged countries on how to deliver better care at a lower cost. And, perhaps most importantly, we can revitalize our personal commitment to become better health professionals and team members by engaging with colleagues around the world who are uncovering new frontiers of our profession.

Through this editorial we would like to encourage the Scandinavian community of family medicine enthusiasts to become involved in this global transition and advancement of our profession. There may be partnerships and projects not mentioned here which could benefit from a Scandinavian network supporting the development of family medicine in Africa.

There are many avenues through which to channel our commitment. As the African proverb reads: “If you want to go fast, go alone. If you want to go far, go together.” We suggest that the Nordic Colleges of General Practitioners collectively join the Primafamed Network Consortium, and that we create a Nordic Group of Family Physicians for Global Health Development under the auspices of the Nordic Federation of General Practice, where the Nordic Congresses can constitute the venue.
